# Genetic variants of *Helicobacter pylori* type IV secretion system components CagL and CagI and their association with clinical outcomes

**DOI:** 10.1186/s13099-017-0165-1

**Published:** 2017-04-21

**Authors:** Hirofumi Ogawa, Akira Iwamoto, Toshihito Tanahashi, Rina Okada, Koji Yamamoto, Shin Nishiumi, Masaru Yoshida, Takeshi Azuma

**Affiliations:** 1 Division of Gastroenterology, Department of Internal Medicine, Kobe University Graduate School of Medicine, 7-5-1 Kusunoki-cho, Chuo-ku, Kobe, Hyogo 650-0017 Japan; 2 Division of Metabolomics Research, Department of Internal Related, Kobe University Graduate School of Medicine, 7-5-1 Kusunoki-cho, Chuo-ku, Kobe, Hyogo 650-0017 Japan; 3 AMED-CREST, AMED, 7-5-1 Kusunoki-cho, Chuo-ku, Kobe, Hyogo 650-0017 Japan; 4 Local Incorporated Administrative Agency, Tokushima Prefecture Naruto Hospital, 32 Muya-cho, Kurosaki Aza Kotani, Naruto, Tokushima 772-0001 Japan

**Keywords:** *Helicobacter pylori*, Whole-genome sequencing, Type IV secretion system, CagL, CagI

## Abstract

**Background:**

*Helicobacter pylori* infection is associated with risk for chronic gastritis (CG), gastric ulcer (GU), duodenal ulcer (DU), and gastric cancer (GC). The *H. pylori* Cag type IV secretion system (TFSS) translocates the virulence factor cytotoxin-associated gene A protein into host cells and plays an important role in initiating gastric carcinogenesis. The CagL and CagI proteins are components of the TFSS. The Arg-Gly-Asp (RGD) motif of CagL, and the six most distal C-terminal amino acids (Ser-Lys-Ile-Ile-Val-Lys, and Ser-Lys-Val-Ile-Val-Lys) of CagL and CagI are essential for TFSS adhesion to host cells. Additionally, the CagL variant Tyr58Glu59 was previously shown to be associated with GC patients.

**Results:**

We isolated 43 *H. pylori* isolates from 17 CG, 8 GU, 8 DU, and 10 GC patients in Southeast Asia. Total DNAs were extracted and sequenced with MiSeq. *H. pylori* strain ATCC 26695, which was isolated from CG patients, was used as a reference. We examined the full sequences of *H. pylori cagL* and *cagI* using whole-genome sequencing (WGS), and analyzed whether single nucleotide variants and amino acid changes (AACs) correlated with adverse clinical outcomes. Three isolates were excluded from the analysis due to *cag*PAI rearrangements. CagL RGD motifs were conserved in 39 isolates (97.5%). CagL-Glu59 and Ile234 in the C-terminal motif were more common in 10 *H. pylori* isolates from GC patients (p < 0.001 and p < 0.05, respectively). When 5 Vietnamese isolates from GC patients were excluded, CagL-Glu59 still remains significant (p < 0.05), but not Ile234. CagL-Tyr58 was seen in only one isolate. The CagI C-terminal motif was completely conserved across all 40 isolates, and there were no significant AACs in CagI.

**Conclusions:**

Using WGS, we analyzed genetic variants in clinical *H. pylori* isolates and identified putative novel and candidate variants in uncharacterized CagL and CagI sequences that are related to gastric carcinogenesis. In particular, CagL-Glu59 has the possible association with GC.

**Electronic supplementary material:**

The online version of this article (doi:10.1186/s13099-017-0165-1) contains supplementary material, which is available to authorized users.

## Background

The infection rate for the Gram-negative bacterium *Helicobacter pylori* is around 50% worldwide [1, 2]. *H. pylori* infection increases the risk of chronic gastritis (CG), gastric ulcer (GU), duodenal ulcer (DU), and gastric cancer (GC). Nevertheless, the exact molecular action to the development of these adverse clinical outcomes remains not well-defined. Especially, in the East Asia, since the infection of cytotoxin-associated gene A (*cagA*) positive *H. pylori* is nearly 100%, their correlation to the different clinical outcomes could not be fully assessed [3–5].

Most *H. pylori* strains (so-called type I strains) contain the *cag* pathogenicity island (*cag*PAI), a chromosomal region that includes about 37,000 bp and 28 genes [3, 4]. Genes encoded in the *cag*PAI allow *H. pylori* bacteria to translocate its major virulence protein cytotoxin associated gene A (CagA) into host gastric epithelial cells using a type-IV secretion system (TFSS) [5, 6]. The role of the *H. pylori* TFSS and CagA translocation was examined in previous sequential studies that showed Src-mediated phosphorylation of CagA tyrosines is important for *H. pylori* virulence [5, 7, 8]. In East Asia in particular, nearly all *H. pylori* infections are CagA positive, which complicates assessment of how clinical *H. pylori* isolates are associated with disease outcomes [9–11]. Moreover, the mechanisms by which *H. pylori* expresses and regulates its TFSS injection apparatus when adapting to human epithelial cell receptors are unclear.

A recent study identified integrin α5β1 expressed on gastric epithelial cells as the putative host receptor for *H. pylori* TFSS [12]. The *H. pylori* CagL protein was found to be an adhesion target on the injected pilus surface for binding to host integrin α5β1 through the CagL Arg-Gly-Asp (RGD) motif [13]. Initial CagL-integrin binding properly induced to locate the bacterial TFSS prior to CagA translocation as well as to activate host tyrosine kinase [12, 14]. This interaction between the *H. pylori* TFSS and host integrin α5β1 can activate the NF-kB proteins and several important pro-inflammatory cytokines that resulted in more adverse clinical outcomes, such as gastric carcinogenesis.

CagI is another *H. pylori* protein, but its function is less clear [12, 15]. CagI has no sequence similarities to any other TFSS components, or to other known proteins [16, 17]. Although an isogenic *cagI* mutant has been examined, there were conflicting reports about whether CagI is required for TFSS function [3, 18]. Based on *H. pylori* transcriptome evidence [19], *cagI* is certainly part of an operon containing *cag*PAI genes involved in the TFSS, but the actual contribution of CagI to clinical phenotypes is unknown.

Here, we used whole-genome sequencing (WGS) to analyze genetic variants of 43 *H. pylori* isolates from patients in Southeast Asia who had different clinical disease. Using the WGS data, we examined whether CagL and/or CagI amino acid changes (AACs) correlated with adverse clinical outcomes such as GC.

## Results

### Characteristics of clinical *H. pylori* isolates

We previously performed WGS on 19 *H. pylori* clinical isolates that we deposited under accession number DRA001250 (see “Methods”). Here we undertook WGS of 24 new clinical *H. pylori* isolates, and analyzed a total of 43 *H. pylori* whole genome sequences (Table 1). The 43 isolates were from 17 chronic gastritis (CG), 8 gastric ulcer (GU), 8 duodenal ulcer (DU) and 10 gastric cancer (GC) patients whose diagnosis was based on endoscopy results. The 43 *H. pylori* isolates we analyzed also had different geographic origins in that 31, 7, and 5 isolates were isolated from Japanese, Chinese, and Vietnamese patients, respectively.

**Table 1:** Characteristics of clinical *H. pylori* isolates and sequencing results

	Strain	Diagnosis	Isolation	Total reads (before trimming)	Total reads (after trimming)	Total consensus length (bp)	Total consensus coverage (%)	Average coverage (fold)	Quality control
1	174	CG	Okinawa	3,004,008	3,003,954	1,534,120	91.98	165.1	Yes
2	177	CG	Okinawa	2,619,844	2,591,582	1,656,888	99.34	101.2	Yes
3	179	CG	Okinawa	2,200,712	2,190,051	1,518,618	91.05	123.6	Yes
4	189	CG	Okinawa	2,010,838	1,989,271	1,651,152	99.00	79.5	No
5	194	CG	Okinawa	6,936,714	6,929,125	1,553,487	93.14	407.5	No
6	S1	GU	Kobe	2,271,944	2,271,917	1,534,031	91.98	144.7	Yes
7	S2	CG	Kobe	5,962,628	5,962,520	1,542,099	92.46	333.4	Yes
8	S4	CG	Kobe	4,640,490	4,640,409	1,536,143	92.10	255.4	Yes
9	S8	CG	Kobe	10,869,516	10,869,208	1,563,806	93.76	669.4	Yes
10	S13	CG	Kobe	3,873,394	3,873,195	1,526,445	91.52	258.2	Yes
11	S16	CG	Kobe	6,782,392	6,782,004	1,555,964	93.29	437.9	Yes
12	S17	GC	Kobe	5,700,038	5,699,682	1,554,277	93.19	318.4	Yes
13	S22	GU	Kobe	6,597,254	6,596,851	1,557,252	93.37	416.5	Yes
14	S23	CG	Kobe	5,064,464	5,040,754	1,514,281	90.79	340.9	Yes
15	S26	CG	Kobe	6,692,038	6,691,708	1,543,795	92.56	432.7	Yes
*16*	*F21*	*GC*	*Fukui*	*3,757,854*	*3,745,628*	*1,511,942*	*90.65*	*263.0*	*Yes*
*17*	*F23*	*CG*	*Fukui*	*5,765,092*	*5,733,420*	*1,514,387*	*90.80*	*387.6*	*Yes*
*18*	*F24*	*DU*	*Fukui*	*4,672,544*	*4,648,960*	*1,527,146*	*91.56*	*323.5*	*Yes*
*19*	*F28*	*DU*	*Fukui*	*7,184,480*	*7,122,238*	*1,537,656*	*92.19*	*365.6*	*Yes*
20	F32	GC	Fukui	5,230,564	5,200,093	1,531,123	91.80	268.2	Yes
21	F44	DU	Fukui	3,887,082	3,846,981	1,663,639	99.75	135.5	Yes
*22*	*F51*	*DU*	*Fukui*	*5,849,012*	*5,816,015*	*1,503,522*	*90.15*	*276.7*	*No*
*23*	*F52*	*DU*	*Fukui*	*4,431,904*	*4,412,519*	*1,531,591*	*91.83*	*310.2*	*Yes*
*24*	*F57*	*GC*	*Fukui*	*6,401,956*	*6,362,361*	*1,526,712*	*91.54*	*405.4*	*Yes*
25	F65	CG	Fukui	4,248,748	4,202,979	1,664,897	99.82	164.9	Yes
*26*	*F75*	*GU*	*Fukui*	*5,186,826*	*5,148,984*	*1,528,778*	*91.66*	*330.2*	*Yes*
27	F79	GU	Fukui	3,036,516	3,036,231	1,521,709	91.24	185.2	Yes
*28*	*F94*	*GU*	*Fukui*	*3,267,624*	*3,247,625*	*1,512,244*	*90.67*	*220.0*	*Yes*
*29*	*F214*	*GU*	*Fukui*	*5,329,874*	*5,285,297*	*1,533,936*	*91.97*	*341.5*	*Yes*
*30*	*F215*	*GU*	*Fukui*	*4,441,566*	*4,411,519*	*1,535,097*	*92.04*	*293.0*	*Yes*
*31*	*F229*	*GU*	*Fukui*	*4,130,184*	*4,105,896*	*1,514,484*	*90.80*	*281.8*	*Yes*
*32*	*HZ2*	*CG*	*Hang Zhou*	*3,953,756*	*3,926,283*	*1,511,585*	*90.63*	*261.8*	*Yes*
*33*	*HZ11*	*CG*	*Hang Zhou*	*5,990,624*	*5,972,745*	*1,517,201*	*90.97*	*422.0*	*Yes*
*34*	*HZ21*	*GC*	*Hang Zhou*	*6,697,176*	*6,676,385*	*1,530,926*	*91.79*	*406.4*	*Yes*
*35*	*HZ34*	*CG*	*Hang Zhou*	*6,245,816*	*6,203,622*	*1,547,241*	*92.77*	*374.0*	*Yes*
*36*	*HZ53*	*DU*	*Hang Zhou*	*6,631,022*	*6,593,830*	*1,556,114*	*93.30*	*402.0*	*Yes*
*37*	*HZ67*	*GC*	*Hang Zhou*	*3,398,652*	*3,388,149*	*1,526,962*	*91.55*	*245.5*	*Yes*
*38*	*HZ82*	*CG*	*Hang Zhou*	*3,984,240*	*3,968,256*	*1,532,373*	*91.88*	*276.1*	*Yes*
*39*	*VN8*	*GC*	*Ho Chi Minh*	*5,876,658*	*5,850,475*	*1,537,665*	*92.19*	*403.1*	*Yes*
*40*	*VN17*	*GC*	*Ho Chi Minh*	*6,810,950*	*6,786,447*	*1,520,811*	*91.18*	*465.3*	*Yes*
*41*	*VN19*	*GC*	*Ho Chi Minh*	*4,071,510*	*4,060,588*	*1,521,512*	*91.23*	*284.2*	*Yes*
*42*	*VN24*	*GC*	*Ho Chi Minh*	*4,241,646*	*4,226,765*	*1,533,944*	*91.97*	*294.1*	*Yes*
*43*	*VN27*	*GC*	*Ho Chi Minh*	*4,315,490*	*4,298,304*	*1,547,804*	*92.80*	*283.4*	*Yes*

Okinawa is an island southwest of the main island of Japan. Kobe and Fukui are located on the main island of Japan. Hang Zhou is located in eastern China and Ho Chi Minh is in southern Vietnam. The 24 strains shown in italics were newly sequenced in this research

*CG* chronic gastritis, *GU* gastric ulcer, *DU* duodenal ulcer, *GC* gastric cancer

### Sequence reads mapping to ATCC 26695 and quality check

The total reads for the 43 *H. pylori* isolates ranged from 1.99 to 10.87 million (Table 1). Sequencing data were mapped to the genome of the *H. pylori* strain ATCC 26695, which was isolated from CG patients, as a reference. Total consensus length (bp) ranged from 1,503,522 to 1,664,897, and total consensus coverage (%) ranged from 90.15 to 99.82%. Average coverage (fold) ranged from 79.5 to 669.4-fold.

Following the initial quality check, we focused on the 28 genes in the *cag*PAI region (Additional file 1: Table S1). Among the 43 isolates, strain ID 189 had lower coverage (under 100-fold) in the *cag*PAI region, strain ID 194 had no genes in the *cag*PAI region, and strain ID F51 carried the *cagA* gene alone. Due to these major sequence differences in the *cag*PAI region, we excluded data for these three isolates, which were all from Japanese patients, such that 40 clinical *H. pylori* isolates were subjected to further analysis. Of these 40 isolates, 15, 8, 7, and 10 were from CG, GU, DU, and GC patients, respectively, and 28, 7, and 5 isolates were derived from Japanese, Chinese, and Vietnamese patients, respectively. CagA motifs of 40 clinical isolates were different (Additional file 2: Table S2).

After the quality check, the average coverage of the remaining 40 isolates ranged from 99.6- to 361.4-fold for *cagL*, and from 105.4- to 416.3-fold for *cagI* (nearly over 100-fold). Consistent with our earlier report, the WGS data in this study had high sequencing coverage, and were of sufficiently high quality to allow detection of SNVs in the *H. pylori* genome [20].

### CagL variants in patients with different clinical disease outcomes

**Fig. 1:**
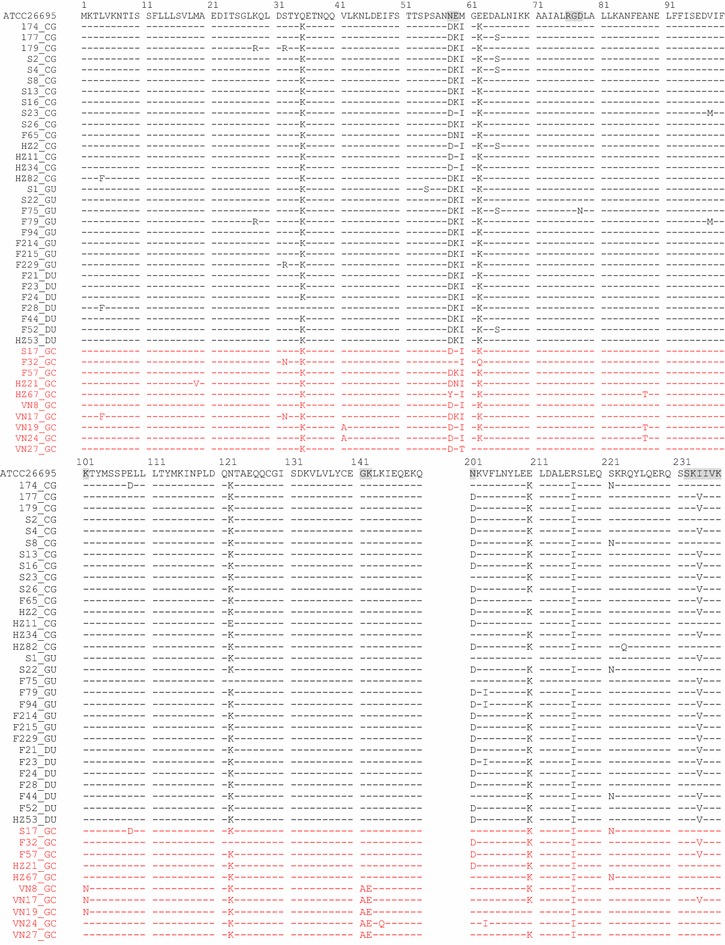
Partial alignment of CagL sequences from 40 isolates from patients with different clinical outcomes. A partial alignment of CagL sequences (aa 1–150 and 201–237) is shown. The 40 clinical isolates included 15 from chronic gastritis (CG), 8 from gastric ulcer (GU), 7 from duodenal ulcer (DU) and 10 from gastric cancer (GC) patients. The amino acid sequence of the *H. pylori* reference strain ATCC 26695 is shown on the *top line*. Tyr58, Glu59, RGD motifs (76–78), Ala141, Glu142, Asn201, and C-terminal motifs of Ser-Lys-Ile-Ile-Val-Lys (232–237) are marked in *grey blocks*. Sequences of 10 isolates from GC patients are indicated in *red*

We translated the CagL nucleotide sequences into amino acid sequences (residues 1–237) with Genomics Workbench 8.5.1, and analyzed CagL variants based on clinical disease outcomes. Table 2 lists CagL variants, and the partial alignments of CagL amino acid changes (AACs) and their locations are shown in Fig. 1. In particular, we characterized AACs present in 10 clinical *H. pylori* isolates derived from GC patients.

**Table 2:** The number of CagL variants in GC and non-GC isolates

Residue	Reference	Variant	GC (n = 10)	(%)	non-GC (n = 30)	(%)	p value (GC versus non-GC)
4	Leu	Phe	1	10	2	6.7	NS
19	Met	Va1	1	10	0	0	NS
28	Lys	Arg	0	0	2	6.7	NS
32	Ser	Arg	0	0	2	6.7	NS
		Asn	2	20	0	0	NS
35	Gln	Lys	10	100	29	96.7	NS
41	Val	Ala	2	20	0	0	NS
54	Pro	Ser	0	0	1	3.3	NS
58	Asn	Asp	8	80	30	100	NS
		Tyr	1	10	0	0	NS
59	Glu	Lys	2	20	25	83.3	0.001
		Asn	1	10	1	3.3	NS
60	Met	Ile	9	90	30	100	NS
		Thr	1	10	0	0	NS
62	Glu	Lys	8	80	30	100	NS
		Gln	1	10	0	0	NS
65	Ala	Ser	0	0	6	20	NS
78	Asp	Asn	0	0	1	3.3	NS
88	Ala	Thr	3	30	0	0	<0.05
98	Val	Met	0	0	2	6.7	NS
101	Lys	Asn	3	30	0	0	<0.05
108	Glu	Asp	1	10	1	3.3	NS
122	Asn	Lys	9	90	28	93.3	NS
		Glu	0	0	1	3.3	NS
141	Gly	Ala	5	50	0	0	<0.001
142	Lys	Glu	5	50	0	0	<0.001
144	Lys	Gln	1	10	0	0	NS
154	Glu	Asp	0	0	2	6.7	NS
158	Thr	Ala	1	10	1	3.3	NS
162	Ala	Thr	0	0	1	3.3	NS
167	Ile	Val	0	0	1	3.3	NS
171	Ala	Thr	0	0	1	3.3	NS
181	Val	Ile	2	20	0	0	NS
201	Asn	Asp	3	30	24	80	<0.05
203	Val	Ile	1	10	3	10	NS
210	Glu	Lys	8	80	27	90	NS
216	Arg	Ile	10	100	28	93.3	NS
221	Ser	Asn	2	20	4	13.3	NS
223	Arg	Gln	0	0	1	3.3	NS
234	Ile	Val	3	30	23	76.7	<0.05

Statistical analysis was performed by Fisher’s exact test. Residue 234 is a part of the C-terminal motif in the distal six amino acids of CagL

*GC* gastric cancer, *NS* not significant

More recently, the CagL variants Tyr58 and/or Glu59 (CagL-Y58E59) was found to occur at significantly higher rates in *H. pylori* isolates from Taiwanese GC patients. CagL-Tyr58Glu59 can induce higher integrin α5β1 expression levels in the upper stomach and increase inflammation in the corpus [21]. Consistent with this report, we found that CagL-Glu59 occurred at a significantly (p < 0.001) higher rate (7/10, 70.0%) in *H. pylori* isolates from GC patients compared to that for 30 *H. pylori* isolates from non-GC patients (4/30, 13.3%). Shown in Table 3, this association between CagL-Glu59 and clinical outcome was still significant with the exception of 5 Vietnamese isolates from GC patients (p < 0.05). The remaining 26 isolates from non-GC patients had Lys59 (K59), and all 15 isolates from DU and GU patients had the CagL-Lys59 variant. In contrast, the reference *H. pylori* strain ATCC 26695 carried CagL-Glu59.

**Table 3:** Seven variants of CagL in GC and non-GC isolates without 5 Vietnamese isolates

Residue	Reference	Variant	GC (n = 5)	(%)	non-GC (n = 30)	(%)	p value (GC versus non-GC)
59	Glu	Lys	1	20.0	25	83.3	<0.05
88	Ala	Thr	1	20.0	0	0.0	NS
101	Lys		0	0	0	0	ND
141	Gly		0	0	0	0	ND
142	Lys		0	0	0	0	ND
201	Asn	Asp	3	60.0	24	80.0	NS
234	Ile	Val	2	40.0	23	76.7	NS

Statistical analysis was performed by Fisher’s exact test. Residue 234 is a part of the C-terminal motif in the distal six amino acids of CagL

*GC* gastric cancer, *NS* not significant, *ND* not determined

Meanwhile, CagL-Tyr58 was present in only one isolate (HZ67) from a GC patient, and its frequency was not significant. Aspartic acid was the most commonly present amino acid at position 58 (Asp58), and occurred in 38 of 40 isolates (95.0%). The remaining isolate (F32) had CagL-Asn58, as did the reference strain ATCC 26695. Only one isolate (HZ67) among the 43 tested had a CagL sequence with both Tyr58 and Glu59.

The C-terminal motifs that include the most distal amino acids of both CagL and CagI are functionally important for the TFSS [22]. In CagL, the sequence of this motif is Ser-Lys-Ile-Ile-Val-Lys (232–237). In this study, we found that Ile234 occurred at a significantly (p = 0.018) higher rate in GC patients (7/10 isolates, 70.0%) relative to that for non-GC patients (23.3%, 7/30). However, Ile234 lost significance with the exception of 5 Vietnamese isolates from GC patients (Table 3). The other five residues showed no significant amino acid sequence differences among the disease outcomes tested.

For CagL, Ala141 and Glu142 variants occurred in all 5 isolates isolated from Vietnamese GC patients. Asp201 had a significantly (p = 0.006) lower frequency in GC patients (3/10 isolates, 30.0%) compared to that for isolates from non-GC patients (24/30, 80.0%). However, Ala141, Glu142, and Asp201 variants lost significance when 5 Vietnamese isolates were excluded (Table 3). Notably, the Arg-Gly-Asp (RGD) motif was well conserved in 39 of 40 isolates (97.5%), but there were no significant differences among disease outcomes.

### CagI variants in patients with different clinical disease outcomes

**Fig. 2:**
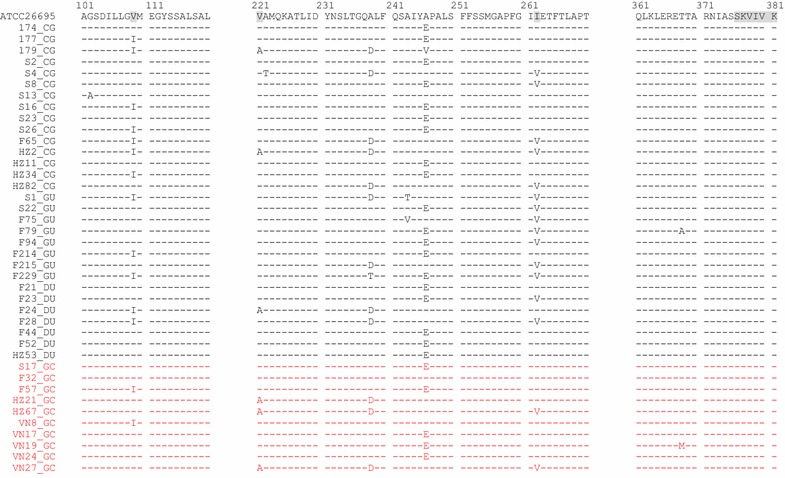
Partial alignment of CagI sequences from 40 isolates from patients with different clinical outcomes. A partial alignment of CagI sequences (aa 101–120, 221–270, and 361–380) is shown. The 40 clinical isolates included 15 from chronic gastritis (CG), 8 from gastric ulcer (GU), 7 from duodenal ulcer (DU), and 10 from gastric cancer (GC) patients. The amino acid sequence of the *H. pylori* reference strain ATCC 26695 is shown on the *top line*. Val109, Ile262, and the Ser-Lys-Val-Ile-Val-Lys (376–381) C-terminal motif are marked by *grey blocks*. The results for 10 isolates from GC patients are indicated in *red*

We also translated CagI nucleotide sequences into amino acid sequences (1–381), and analyzed rates and locations of CagI variants based on clinical disease outcomes (Table 4; Fig. 2).

**Table 4:** The number of CagI variants in GC and non-GC isolates

Residue	Reference	Variant	GC (n = 10)	(%)	non-GC (n = 30)	(%)	p value (GC versus non-GC)
1	Val	Glu	1	10	0	0	NS
2	Lys	Met	1	10	0	0	NS
3	Cys	Phe	1	10	0	0	NS
		Tyr	0	0	1	3.3	NS
5	Leu	Lys	1	10	0	0	NS
6	Ser	His	1	10	0	0	NS
7	Ile	Val	0	0	1	3.3	NS
10	Phe	Phe	0	0	1	3.3	NS
15	Gly	Ser	0	0	1	3.3	NS
17	Ser	Phe	9	90	28	93.3	NS
21	Thr	Thr	3	30	7	23.3	NS
22	Glu	Gly	1	10	8	26.7	NS
23	Val	Ala	8	80	26	86.7	NS
29	Pro	Ser	9	90	28	93.3	NS
36	Ala	Val	0	0	1	3.3	NS
40	Ala	Val	0	0	1	3.3	NS
57	Ser	Asn	1	10	2	6.7	NS
65	Ala	Val	1	10	1	3.3	NS
70	Glu	Gln	5	50	25	83.3	NS
78	Met	Ile	9	90	27	90	NS
94	Gly	Ser	10	100	30	100	NS
102	Gly	Ala	0	0	1	3.3	NS
109	Val	Ile	2	20	12	40	NS
125	Lys	Asn	9	90	27	90	NS
152	Ile	Met	7	70	19	63.3	NS
165	Glu	Gln	1	10	0	0	NS
179	Thr	Ala	1	10	0	0	NS
182	Glu	Ala	0	0	1	3.3	NS
190	Ser	Asn	0	0	1	3.3	NS
195	Ala	Thr	0	0	4	13.3	NS
196	Gln	Lys	8	80	26	86.7	NS
203	Ile	Val	1	10	0	0	NS
207	Ala	Thr	0	0	1	3.3	NS
213	Lys	Glu	1	10	1	3.3	NS
214	Gly	Asp	0	0	1	3.3	NS
221	Val	Ala	3	30	3	10	NS
222	Ala	Thr	0	0	1	3.3	NS
238	Ala	Asp	3	30	9	30	NS
		Thr	0	0	1	3.3	NS
243	Ala	Thr	0	0	1	3.3	NS
		Val	0	0	1	3.3	NS
246	Ala	Glu	5	50	19	63.3	NS
		Val	0	0	1	3.3	NS
262	Ile	Val	2	20	14	46.7	NS
294	Met	Lys	9	90	30	100	NS
304	Ser	Asn	9	90	30	100	NS
319	Gly	Glu	9	90	30	100	NS
346	Asn	Ser	0	0	1	3.3	NS
368	Thr	Ala	0	0	1	3.3	NS
		Met	1	10	0	0	NS

Statistical analysis was performed by Fisher’s exact test

*GC* gastric cancer, *NS* not significant

As with CagL, the C-terminal motif of Ser-Lys-Val-Ile-Val-Lys (376–381) in CagI is functionally essential for the TFSS. In our analysis, all 40 *H. pylori* isolates from both GC and non-GC patients had the same motif, which had a completely conserved sequence.

Valine at CagI amino acid residue 109 (Val109) was frequent in *H. pylori* isolates from both GC patients (8/10, 80.0%) and non-GC patients (18/30, 60.0%). Isoleucine at position 262 (Ile262) was similarly frequent in GC patients (8/10, 80%) and non-GC patients (16/30, 53.3%), and the difference in rates was not significant. There were no other AACs associated with clinical outcome in the CagI sequence.

### Phylogenetic implications of *H. pylori* CagL and CagI diversity

**Fig. 3:**
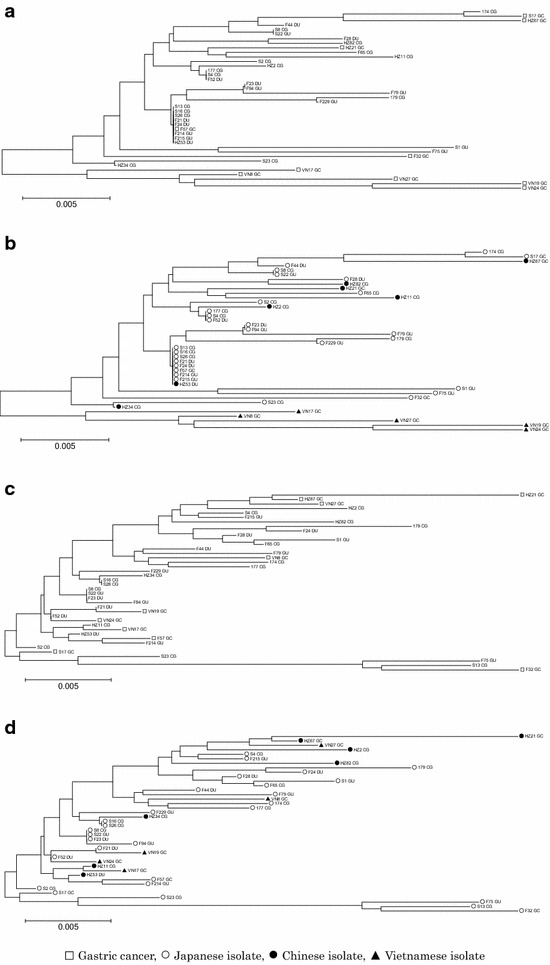
Phylogenetic tree of 40 clinical isolates based on CagL and CagI sequences. Neighbor-Joining tree analysis of concatenated CagL (**a**, **b**) and CagI (**c**, **d**) sequences for 40 isolates is shown. Each Neighbor-Joining method tree was made using MEGA7 software. *Open square*, *open circle*, *filled circle*, and *filled triangle* symbols correspond to isolates derived from gastric cancer patients, Japanese isolates, Chinese isolates, and Vietnamese isolates, respectively

Phylogenetic trees were conducted using MEGA7 [23]. In general, CagL sequences showed no characteristic clusters around disease outcomes (Fig. 3a), although there was a cluster among the five Vietnamese isolates (Fig. 3b). Meanwhile, CagI sequences had no characteristic clusters for either region or disease outcome (Fig. 3c, d).

## Discussion

Using the advantages provided by whole-genome sequencing (WGS), we analyzed candidate and novel variants of CagL and CagI proteins in 40 clinical *H. pylori* isolates from patients in Southeast Asia. We showed that CagL from *H. pylori* isolates derived from GC patients carried several specific amino acid changes (AACs), but we detected no significant changes in the CagI amino acid sequence.

Whole-genome sequencing technology was recently applied to clarify the pathogenicity and evolution of *H. pylori*, as well as to identify its virulence factors [24, 25]. Using WGS, we and others detected potential mutations throughout the *H. pylori* genome and identified variants when sequence changes were present [20, 24, 25]. Here, we used WGS technology to detect novel variants in uncharacterized *cag*PAI genes associated with *H. pylori* pathogenicity.

*cag*PAI is a 37 kb segment of *H. pylori* DNA that contains 28 genes [3, 4], and is found in about 60% of Western isolates, whereas nearly all East Asian isolates are *cag*PAI positive [26]. We analyzed *cag*PAI integrity and showed the rearrangement of this island in three Japanese isolates (189, 194 and F51). Although *cag*PAI was most intact in Japanese isolates, it was disrupted in isolates isolated throughout the world [27]. Since the pathogenic role of the *cag*PAI is well defined as a whole or in part, we excluded the three isolates that had *cag*PAI rearrangements.

Several Cag proteins have been detected as constituents of the *H. pylori cag* TFSS apparatus and have important roles in CagA translocation [14, 15, 22]. The CagL and CagI proteins have been previously characterized [16, 17], so in this study we used WGS to screen 40 clinical *H. pylori* isolates for CagL and CagI variants, and analyzed the relationship between amino acid sequence and clinical outcomes. Consistent with a previous report [21], we detected complete RGD motifs in CagL sequences from all isolates. These data highlight the importance of the RGD motif for CagL function in the TFSS. We also checked whether other AACs in CagL and CagI were correlated with clinical outcomes.

We further confirmed that the frequency of the candidate variant CagL-Glu59 in GC patients significantly differed from that seen for isolates from non-GC patients. This association of Glu59 was still significant with the exception of 5 Vietnamese isolates, which was the half of all GC isolates (5/10). However, the frequency of Tyr58 was not significantly different, which is in contrast to a previous study that showed the CagL-Tyr58Glu59 variants were more common in *H. pylori* isolates from GC patients [21]. CagL-Tyr58Glu59 variants have strong binding affinity for integrin α5β1 and also promote increased expression of this integrin, and significantly enhances CagA translocation and phosphorylation relative to wild type CagL [28]. However, these results contrasted with those shown by Tegtmeyer et al. [29]. Our data support the importance of CagL-Glu59 variant, and imply that Glu59 could be incorporated into strategies to screen clinical *H. pylori* isolates. However, the current study is rather small and limited to the patients in Southeast Asia. These results require validation with larger isolates in Southeast Asia and the other samples in Western countries.

The C-terminal motif in CagL and CagI consisting of six amino acids (Ser-Lys-Ile-Ile-Val-Lys, and Ser-Lys-Val-Ile-Val-Lys, respectively) is important for TFSS function [22]. However, whether these sequences were conserved among genomes of clinical *H. pylori* isolates was unclear. Here, we showed that the CagI C-terminal motif was completely conserved. Although the CagL C-terminal motif was also well conserved, we found a significant difference at position 234 of CagL among *H. pylori* isolates derived from GC and non-GC patients. However, Ile234 lost significance with the exception of 5 Vietnamese isolates from GC patients. Future studies on additional *H. pylori* isolates could validate whether CagL-Ile234 could serve as a marker that indicates an increased risk for gastric carcinogenesis.

## Conclusions

We analyzed genetic variants of *H. pylori* using WGS, which has significant advantages over other approaches that examine only a fraction of the genome at any one time. WGS identified several putative novel variants of CagL and CagI sequences from previously uncharacterized *H. pylori* isolates. These variants, particularly in CagL-Glu59, have the possible effect on the TFSS activity and the relevance with clinical outcomes.

## Methods

### *H. pylori* samples

Forty-three *H. pylori* clinical isolates were obtained from gastric epithelium biopsy tissues taken during upper gastroduodenal endoscopy procedures performed at Okinawa Prefectural Chubu Hospital, Kobe University Hospital, and Fukui University Hospital in Japan, as well as Zhejiang University Hospital in China and Cho Ray Hospital in Vietnam. All patients gave written informed consent for use of their samples in this study, which was performed according to the principles of the Declaration of Helsinki. The major reference strain, ATCC 26695 (NC_000915), was isolated from CG patients in the United Kingdom [30], and its sequence served as the reference sequence.

### *H. pylori* culture

Gastric biopsy specimens were first inoculated onto trypticase soy agar II (TSA-II)-5% sheep blood plates (Becton, Dickinson and Company: BD) and cultured under microaerophilic conditions (O_2_ 5%; CO_2_ 5%; N_2_ 90%) at 37 °C for 3–5 days. Then, one colony was picked from each primary culture plate, and seeded onto a Columbia *Helicobacter pylori* agar plate containing vancomycin (10 mg/l), trimethoprim (5 mg/l), amphotericin B (5 mg/l), and polymyxin B (2500 units/l), and cultured under the same conditions. A colony was picked from this second plate, seeded onto a TSA-II plate, and cultured under the same conditions. Several colonies were picked from the third plate, transferred into Brucella Broth medium (2 ml) containing 10% fetal calf serum, and cultured for 18 h under the same conditions.

A portion of each culture was stored at −80 °C in 0.01 M phosphate-buffered saline (PBS), pH 7.4, containing 20% glycerol. *H. pylori* DNA was extracted from bacterial pellets prepared from liquid cultures using the protease–phenol–chloroform method. The extracted DNA was suspended in 100 μl distilled water and stored at 4 °C.

### Whole-genome sequencing (WGS)

Total DNA of *H. pylori* isolated from patients and the reference strain ATCC 26695 were sequenced. The bacterial DNA concentration of each sample was measured with a Qubit dsDNA HS assay kit (Q32851; Invitrogen, Carlsbad, CA) and the concentration of each sample was between 250 and 320 pg/μl.

A DNA library of *H. pylori* isolates was prepared using a Nextera XT DNA Sample Prep Kit (Illumina, Carlsbad, CA), which was used according to the manufacturer’s instructions to uniformly shear the DNA into 500 bp fragments and add unique adapter sequences to the fragments. The resulting DNA library was run on a MiSeq sequencer (Illumina) with a reagent kit (300 cycle, paired-end). Fluorescence images were analyzed using MiSeq Control Software, and FASTQ-formatted sequence data were generated using MiSeq Reporter Analysis.

### Sequence read mapping and single nucleotide variant (SNV) detection

For the analyzed DNA sequence data, read qualities having a Q30 value above 80% were selected according to recommendations by Illumina. After a quality check and data trimming, the sequence reads were assembled with Genomics Workbench 8.5.1 (CLC bio, Aarhus, Denmark). The read mapping module was termed as CLC Assembly Cell 4.0, which was based on an uncompressed Suffix-Array representing the entire reference genome in a single data structure (White paper on CLC read mapper; October 10, 2012). Sequence reads were mapped against the ATCC 26695 genome (NC_000915) as a reference, and single nucleotide variants (SNVs) were identified with Fixed Ploidy Variant Detection modules with default parameters and minor modifications to the mapping algorithm. Variant detection of the software was set to 1.

To exclude false-positive variants that resulted from sequencing errors, we selected variants that were present in >90.0% of mapped reads with a minimum coverage of 100. Insertions, deletions, and successive multi nucleotide variants were also excluded due to the previously reported complexity involved in detecting true variants [18].

### Phylogenetic analysis

We constructed a phylogenetic tree from CagL and CagI sequences of *H. pylori* isolates using Molecular Evolutionary Genetics Analysis version 7.0 (MEGA7) [23]. Evolutionary history was inferred using the Neighbor-Joining tree [31]. Trees were drawn to scale, wherein branch lengths are shown in the same units as those of the evolutionary distances used to infer the phylogenetic tree. The analysis involved 40 isolates, and the CagL and CagI sequences included 237 and 381 amino acids, respectively.

### Statistical analysis

Differences in the number of amino acid changes (AAC) in CagL and CagI in clinical outcomes and regions in Southeast Asia were compared using the Fisher’s exact test. A difference associated with a p value <0.05 was considered to be significant. The SPSS statistical software package version 23.0.0.0 (SPSS, Inc., Chicago, IL) was used for all statistical analyses.

### Nucleotide sequence accession number

Sequence reads of 19 Japanese clinical isolates and ATCC 26695 were previously deposited in the DNA Data Bank of Japan Sequence Read Archive (http://www.ddbj.nig.ac.jp/index-e.html) under accession number DRA001250. Sequence reads of 5 Vietnamese clinical isolates were deposited under accession number DRA002946, whereas 7 Chinese isolates and an additional 12 Japanese isolates were deposited under DRA004713.

## Additional files

**Additional file 1: Table S1.** Average coverage (fold) of 28 *cag*PAI genes in 43 clinical *H. pylori* isolates mapped to the ATCC26695 sequence.

**Additional file 2: Table S2.** CagA motifs of 40 clinical isolates.

## Abbreviations

*H. pylori*: *Helicobacter pylori*
WGS: whole-genome sequencing
SNVs: single nucleotide variants
AACs: amino acid changes
TFSS: type-IV secretion system
cag: cytotoxin-associated gene
CG: chronic gastritis
GU: gastric ulcer
DU: duodenal ulcer
GC: gastric cancer
