# Supplementary material for: Genetic variants of Helicobacter pylori type IV secretion system components CagL and CagI and their association with clinical outcomes
Source: Gut Pathog. 2017 Apr 21;9:21. doi: 10.1186/s13099-017-0165-1 (PMC5399799; doi:10.1186/s13099-017-0165-1)
Supplement: Supplementary file 1 — Additional file 1: Table S1. Average coverage (fold) of 28 cagPAI genes in 43 clinical H. pylori isolates mapped to the ATCC26695 sequence. [file 13099_2017_165_MOESM1_ESM.docx]

**Supplemental Table 1. Average coverage (fold) of 28 *cag*PAI genes in 43 clinical *H. pylori* isolates mapped to the ATCC26695 sequence**

| Gene name | | *cagζ* | *cagε* | *cagδ* | *cagγ* | *cagβ* | *cagα* | *cagZ* | *cagY* | *cagX* | *cagW* | *cagV* | *cagU* | *cagT* | *cagS* | *cagQ* | *cagP* | *cagM* | *cagN* | *cagL* | *cagI* | *cagH* | *cagG* | *cagF* | *cagE* | *cagD* | *cagC* | *cagA* |  |
| --- | --- | --- | --- | --- | --- | --- | --- | --- | --- | --- | --- | --- | --- | --- | --- | --- | --- | --- | --- | --- | --- | --- | --- | --- | --- | --- | --- | --- | --- |
| Locus tag | | HP0520 | HP0521* | HP0522 | HP0523 | HP0524 | HP0525 | HP0526 | HP0527 | HP0528 | HP0529 | HP0530 | HP0531 | HP0532 | HP0534 | HP0535 | HP0536 | HP0537 | HP0538 | HP0539 | HP0540 | HP0541 | HP0542 | HP0543 | HP0544 | HP0545 | HP0546 | HP0547 | HP0548 |
| Region length | | 348 | 243 | 1446 | 510 | 2247 | 993 | 600 | 5784 | 1569 | 1608 | 759 | 657 | 843 | 591 | 381 | 345 | 1131 | 921 | 714 | 1146 | 1113 | 429 | 807 | 2952 | 624 | 348 | 3561 | 828 |
| 1 | 174 | 101 | 0 | 128 | 134 | 131 | 140 | 113 | 118 | 122 | 121 | 113 | 111 | 146 | 128 | 96 | 97 | 98 | 118 | 112 | 136 | 161 | 132 | 104 | 116 | 105 | 118 | 99 | 0 |
| 2 | 177 | 92 | 3 | 117 | 108 | 125 | 139 | 95 | 84 | 103 | 111 | 92 | 110 | 135 | 125 | 79 | 118 | 71 | 84 | 100 | 108 | 144 | 147 | 97 | 100 | 88 | 112 | 68 | 3 |
| 3 | 179 | 129 | 0 | 149 | 110 | 144 | 167 | 105 | 143 | 147 | 117 | 123 | 96 | 177 | 132 | 96 | 118 | 107 | 142 | 129 | 152 | 153 | 125 | 123 | 146 | 99 | 125 | 119 | 0 |
| 4 | 189 | 37 | 2 | 51 | 36 | 73 | 51 | 29 | 23 | 11 | 10 | 10 | 12 | 13 | 13 | 7 | 13 | 10 | 8 | 9 | 9 | 13 | 11 | 10 | 9 | 7 | 11 | 6 | 26 |
| 5 | 194 | 0 | 0 | 0 | 0 | 0 | 0 | 0 | 0 | 0 | 0 | 0 | 0 | 0 | 0 | 0 | 0 | 0 | 0 | 0 | 0 | 0 | 0 | 0 | 0 | 0 | 1 | 0 | 0 |
| 6 | S1 | 90 | 0 | 112 | 96 | 103 | 130 | 92 | 103 | 122 | 97 | 88 | 85 | 121 | 124 | 62 | 108 | 86 | 105 | 103 | 105 | 125 | 111 | 118 | 107 | 89 | 88 | 88 | 0 |
| 7 | S2 | 274 | 0 | 428 | 390 | 427 | 500 | 296 | 341 | 403 | 338 | 283 | 321 | 423 | 400 | 233 | 334 | 267 | 349 | 350 | 416 | 496 | 407 | 332 | 378 | 298 | 345 | 172 | 0 |
| 8 | S4 | 103 | 0 | 164 | 135 | 150 | 162 | 114 | 113 | 141 | 112 | 105 | 105 | 147 | 145 | 81 | 130 | 107 | 126 | 133 | 139 | 189 | 165 | 124 | 149 | 134 | 144 | 137 | 0 |
| 9 | S8 | 329 | 1 | 414 | 371 | 386 | 431 | 274 | 269 | 310 | 300 | 274 | 280 | 374 | 358 | 226 | 298 | 282 | 314 | 324 | 355 | 443 | 375 | 351 | 368 | 322 | 358 | 327 | 0 |
| 10 | S13 | 117 | 107 | 166 | 136 | 147 | 158 | 108 | 97 | 117 | 116 | 100 | 103 | 130 | 123 | 95 | 117 | 95 | 114 | 121 | 123 | 162 | 148 | 126 | 137 | 118 | 134 | 134 | 0 |
| 11 | S16 | 191 | 0 | 267 | 240 | 262 | 290 | 195 | 185 | 243 | 210 | 163 | 198 | 238 | 237 | 148 | 201 | 167 | 207 | 214 | 235 | 293 | 266 | 220 | 228 | 220 | 224 | 212 | 0 |
| 12 | S17 | 165 | 1 | 277 | 255 | 275 | 336 | 199 | 223 | 260 | 245 | 209 | 221 | 280 | 270 | 161 | 219 | 184 | 235 | 248 | 256 | 328 | 287 | 219 | 231 | 181 | 219 | 197 | 0 |
| 13 | S22 | 189 | 1 | 253 | 230 | 226 | 268 | 177 | 168 | 193 | 181 | 146 | 165 | 232 | 202 | 151 | 190 | 161 | 195 | 192 | 220 | 276 | 232 | 220 | 217 | 204 | 234 | 201 | 0 |
| 14 | S23 | 156 | 0 | 316 | 232 | 265 | 286 | 144 | 256 | 250 | 176 | 169 | 135 | 285 | 181 | 89 | 121 | 100 | 212 | 232 | 247 | 364 | 199 | 89 | 213 | 110 | 227 | 188 | 0 |
| 15 | S26 | 188 | 0 | 264 | 239 | 258 | 287 | 194 | 183 | 239 | 208 | 161 | 195 | 237 | 234 | 147 | 199 | 166 | 204 | 212 | 233 | 291 | 264 | 217 | 226 | 217 | 221 | 210 | 0 |
| 16 | F21 | 194 | 0 | 246 | 230 | 214 | 237 | 166 | 170 | 217 | 192 | 180 | 180 | 209 | 210 | 157 | 206 | 166 | 193 | 216 | 199 | 267 | 193 | 172 | 191 | 167 | 188 | 148 | 0 |
| 17 | F23 | 236 | 0 | 378 | 306 | 327 | 366 | 241 | 260 | 327 | 271 | 253 | 272 | 327 | 306 | 212 | 311 | 224 | 300 | 278 | 312 | 387 | 333 | 262 | 268 | 243 | 289 | 210 | 0 |
| 18 | F24 | 131 | 0 | 200 | 165 | 186 | 219 | 133 | 139 | 182 | 149 | 116 | 134 | 173 | 165 | 113 | 175 | 119 | 150 | 145 | 159 | 223 | 191 | 141 | 160 | 139 | 148 | 138 | 0 |
| 19 | F28 | 223 | 0 | 314 | 259 | 305 | 365 | 194 | 240 | 306 | 258 | 191 | 239 | 321 | 288 | 150 | 234 | 169 | 243 | 301 | 307 | 376 | 291 | 229 | 250 | 222 | 262 | 213 | 0 |
| 20 | F32 | 127 | 0 | 204 | 174 | 183 | 206 | 121 | 148 | 159 | 145 | 136 | 129 | 194 | 163 | 133 | 122 | 117 | 142 | 170 | 174 | 216 | 171 | 144 | 156 | 144 | 138 | 121 | 0 |
| 21 | F44 | 112 | 2 | 161 | 140 | 158 | 177 | 128 | 123 | 161 | 153 | 133 | 135 | 179 | 153 | 123 | 135 | 112 | 128 | 134 | 156 | 180 | 156 | 131 | 135 | 124 | 136 | 101 | 5 |
| 22 | F51 | 0 | 0 | 0 | 0 | 0 | 0 | 0 | 0 | 0 | 0 | 0 | 0 | 0 | 0 | 0 | 0 | 0 | 0 | 0 | 0 | 0 | 0 | 0 | 0 | 0 | 0 | 197 | 0 |
| 23 | F52 | 165 | 0 | 229 | 196 | 211 | 246 | 189 | 168 | 239 | 175 | 161 | 166 | 210 | 210 | 140 | 194 | 135 | 198 | 196 | 211 | 260 | 214 | 185 | 196 | 159 | 212 | 157 | 0 |
| 24 | F57 | 250 | 0 | 342 | 341 | 353 | 412 | 279 | 269 | 333 | 277 | 265 | 248 | 351 | 308 | 224 | 317 | 228 | 293 | 288 | 343 | 387 | 304 | 257 | 288 | 263 | 283 | 228 | 0 |
| 25 | F65 | 134 | 4 | 186 | 165 | 206 | 221 | 142 | 144 | 176 | 169 | 140 | 167 | 213 | 187 | 130 | 186 | 127 | 147 | 162 | 179 | 248 | 220 | 145 | 146 | 133 | 162 | 118 | 6 |
| 26 | F75 | 290 | 0 | 462 | 429 | 416 | 523 | 331 | 377 | 421 | 349 | 352 | 337 | 460 | 427 | 285 | 351 | 292 | 376 | 361 | 412 | 539 | 444 | 386 | 373 | 349 | 400 | 167 | 0 |
| 27 | F79 | 87 | 0 | 148 | 153 | 143 | 159 | 106 | 94 | 117 | 116 | 106 | 103 | 121 | 113 | 91 | 93 | 73 | 86 | 101 | 127 | 153 | 150 | 122 | 109 | 89 | 113 | 121 | 0 |
| 28 | F94 | 115 | 0 | 181 | 163 | 170 | 175 | 142 | 128 | 163 | 123 | 124 | 114 | 167 | 136 | 87 | 128 | 117 | 145 | 139 | 156 | 183 | 150 | 125 | 132 | 125 | 121 | 128 | 0 |
| 29 | F214 | 191 | 0 | 290 | 265 | 260 | 331 | 221 | 203 | 285 | 232 | 194 | 199 | 288 | 242 | 183 | 233 | 170 | 226 | 236 | 278 | 327 | 253 | 213 | 228 | 174 | 256 | 182 | 0 |
| 30 | F215 | 152 | 0 | 250 | 221 | 224 | 307 | 192 | 173 | 239 | 189 | 173 | 169 | 259 | 203 | 139 | 214 | 144 | 198 | 217 | 224 | 250 | 221 | 174 | 194 | 181 | 230 | 162 | 0 |
| 31 | F229 | 144 | 0 | 205 | 196 | 186 | 222 | 137 | 141 | 216 | 148 | 144 | 138 | 197 | 165 | 134 | 150 | 128 | 170 | 173 | 193 | 212 | 186 | 158 | 169 | 173 | 192 | 147 | 0 |
| 32 | HZ2 | 110 | 0 | 241 | 193 | 201 | 237 | 151 | 193 | 184 | 147 | 144 | 101 | 231 | 147 | 74 | 97 | 93 | 164 | 175 | 223 | 273 | 163 | 96 | 158 | 138 | 197 | 143 | 0 |
| 33 | HZ11 | 260 | 0 | 384 | 278 | 326 | 378 | 247 | 268 | 341 | 269 | 229 | 231 | 357 | 243 | 195 | 264 | 194 | 269 | 303 | 365 | 466 | 308 | 198 | 272 | 245 | 321 | 248 | 0 |
| 34 | HZ21 | 213 | 0 | 377 | 352 | 339 | 380 | 233 | 270 | 326 | 275 | 282 | 253 | 361 | 289 | 148 | 224 | 196 | 302 | 328 | 348 | 454 | 291 | 204 | 264 | 233 | 294 | 251 | 0 |
| 35 | HZ34 | 196 | 0 | 338 | 296 | 310 | 386 | 295 | 266 | 328 | 283 | 268 | 297 | 345 | 299 | 194 | 280 | 215 | 271 | 283 | 322 | 420 | 344 | 276 | 270 | 243 | 278 | 229 | 0 |
| 36 | HZ53 | 197 | 1 | 364 | 346 | 368 | 390 | 253 | 284 | 350 | 262 | 240 | 244 | 374 | 311 | 215 | 282 | 210 | 285 | 301 | 337 | 423 | 357 | 249 | 265 | 263 | 297 | 306 | 0 |
| 37 | HZ67 | 95 | 0 | 139 | 117 | 123 | 145 | 104 | 121 | 148 | 107 | 97 | 96 | 132 | 123 | 99 | 124 | 98 | 138 | 126 | 126 | 180 | 136 | 112 | 118 | 116 | 123 | 120 | 0 |
| 38 | HZ82 | 107 | 0 | 165 | 160 | 155 | 179 | 141 | 141 | 157 | 117 | 110 | 114 | 138 | 124 | 101 | 131 | 93 | 127 | 120 | 131 | 179 | 145 | 126 | 136 | 120 | 148 | 156 | 0 |
| 39 | VN8 | 134 | 0 | 195 | 197 | 236 | 232 | 149 | 115 | 115 | 119 | 82 | 96 | 104 | 95 | 52 | 79 | 67 | 90 | 106 | 148 | 202 | 160 | 131 | 149 | 126 | 146 | 143 | 0 |
| 40 | VN17 | 285 | 0 | 409 | 362 | 382 | 482 | 255 | 274 | 319 | 291 | 257 | 303 | 367 | 337 | 179 | 298 | 206 | 290 | 314 | 391 | 504 | 336 | 288 | 322 | 265 | 299 | 255 | 0 |
| 41 | VN19 | 152 | 0 | 257 | 212 | 240 | 292 | 188 | 218 | 235 | 197 | 186 | 170 | 270 | 266 | 126 | 187 | 145 | 225 | 198 | 242 | 308 | 211 | 204 | 208 | 151 | 172 | 163 | 0 |
| 42 | VN24 | 204 | 0 | 226 | 229 | 237 | 244 | 199 | 258 | 225 | 197 | 171 | 178 | 219 | 208 | 166 | 170 | 176 | 208 | 212 | 235 | 255 | 225 | 167 | 200 | 154 | 165 | 164 | 0 |
| 43 | VN27 | 168 | 1 | 217 | 192 | 203 | 247 | 189 | 189 | 196 | 175 | 139 | 173 | 206 | 193 | 98 | 166 | 145 | 174 | 184 | 189 | 228 | 195 | 175 | 180 | 172 | 176 | 155 | 0 |

Locus tag is based on ATCC26695 genome annotation.
