# Supplementary material for: Genetic variants of Helicobacter pylori type IV secretion system components CagL and CagI and their association with clinical outcomes
Source: Gut Pathog. 2017 Apr 21;9:21. doi: 10.1186/s13099-017-0165-1 (PMC5399799; doi:10.1186/s13099-017-0165-1)
Supplement: Supplementary file 2 — Additional file 2: Table S2. CagA motifs of 40 clinical isolates. [file 13099_2017_165_MOESM2_ESM.docx]

| Strain | CagA motif | Diagnosis | Strain | CagA motif | Diagnosis | Strain | CagA motif | Diagnosis |
| --- | --- | --- | --- | --- | --- | --- | --- | --- |
| 174 | ABD | CG | F21 | ABD | GC | HZ2 | ABD | CG |
| 177 | ABD | CG | F23 | ABD | CG | HZ11 | ABD | CG |
| 179 | ABD | CG | F24 | ABD | DU | HZ21 | ABD | GC |
|  |  |  | F28 | ABD | DU | HZ34 | ABD | CG |
| S1 | AD | GU | F32 | ABD | GC | HZ53 | AC | DU |
| S2 | ABD | CG | F44 | ABD | DU | HZ67 | ABD | GC |
| S4 | AD | CG | F52 | ABD | DU | HZ82 | ABD | CG |
| S8 | AD | CG | F57 | ABD | GC |  |  |  |
| S13 | ABD | CG | F65 | ABB | CG | VN8 | ABD | GC |
| S16 | AD | CG | F75 | AD | GU | VN17 | ABD | GC |
| S17 | ABD | GC | F79 | AC | GU | VN19 | AD | GC |
| S22 | AD | GU | F94 | ABD | GU | VN24 | ABD | GC |
| S23 | ABD | CG | F214 | ABD | GU | VN27 | ABD | GC |
| S26 | AD | CG | F215 | ABD | GU |  |  |  |
|  |  |  | F229 | ABD | GU |  |  |  |

**Supplementary Table 2. CagA motifs of 40 clinical isolates**

CG; chronic gastritis, GU; gastric ulcer, DU; duodenal ulcer, and GC; gastric cancer. Total reads after trimming were mapped to the reference nucleotide of F32 (NC_017366) calculated with Genomic Workbench 8.5.1.
